# Behavioral activation therapy for depression and anxiety in cancer patients: a case series study

**DOI:** 10.1186/s13030-019-0151-6

**Published:** 2019-04-29

**Authors:** Takatoshi Hirayama, Yuko Ogawa, Yuko Yanai, Shin-ichi Suzuki, Ken Shimizu

**Affiliations:** 1 Department of Psycho-Oncology, National Cancer Center Hospital, 5-1-1 Tsukiji, Chuo-ku, Tokyo 104-0045 Japan; 2 Faculty of Human Sciences, Waseda University, 2-579-15 Mikajima, Tokorozawa-city, Saitama, Tokyo 359-1192 Japan

**Keywords:** Behavioral activation therapy, Cancer, Depression, Anxiety, Adjustment disorders, Psychotherapy

## Abstract

**Background:**

Behavioral activation therapy (BAT) directly addresses activities that individuals value most highly, and may be easily applicable to cancer patients. However, there is no established evidence of the use of BAT in this population. In this study, we examined the possibility of a BAT program for depression and anxiety in cancer patients.

**Case presentation:**

We retrospectively reviewed the medical records of cancer patients with each of the following characteristics: 1) were outpatients or inpatients visiting the psycho-oncology division of the National Cancer Center Hospital in Japan; 2) met criteria for Major Depressive Disorder or Adjustment Disorders; and 3) participated in a BAT program. The primary outcome was the program completion percentage. Secondary outcomes were self-reported depression severity (Patient Health Questionnaire-9 (PHQ-9) score), anxiety disorder status (Generalized Anxiety Disorder-7 (GAD-7) score), and clinical improvement (Clinical Global Impression-Improvement (CGI-I) score) after the program. We analyzed both depression and anxiety by the matched paired t-test. Ten patients participated in the program, and nine completed it. One dropped out due to cognitive impairment secondary to brain metastasis. Both the PHQ-9 scores (pre: 14.4 (SD, 6.1); post: 5.1 (SD, 5.8)) and the GAD-7 scores (pre: 11.9 (SD, 4.9); post: 4.7 (SD, 5.5)) significantly improved after the program (PHQ-9: *P* = 0.0014; GAD-7: *P* = 0.0004). CGI-I scores ranged from 1 to 3, and all subjects except the patient who dropped out improved clinically. Among the ten patients, three distinctive cases could be observed as follows. Case 1; a 45-year-old housewife with breast cancer who did not agree to take antidepressants because of concerns about the side effects achieved remission without antidepressants and began to live an active life. Case 4; a 66-year-old housewife was so shocked after endometrial cancer diagnosis that she was absent-minded and her compliance with the assigned homework was poor, therefore, her depression did not improve much. Case 9; a 62-year-old man with laryngeal cancer who had recurrent anxiety. Increased business activity, on which he put great value, gradually allowed him to be able to live his life actively without concerns.

**Conclusions:**

This study suggests that BAT would be effective for the depression and anxiety of cancer patients.

## Background

Psychiatric disorders such as major depression and adjustment disorders are reported to occur in 30–40% of patients in the oncological setting [1]. Most depressive cancer patients have mild or reactive depression [2]. Pharmacotherapy is recommended for patients with moderate or severe depression and chronic physical health problems, while psychotherapy is preferred in cases of persistent subthreshold depressive symptoms or mild depression [3]. Furthermore, a meta-analysis reported that psychotherapy was effective for cancer patients with depressive symptoms, including those with adjustment disorders [4]. Therefore, most cancer patients with depressive symptoms are candidates for psychotherapy rather than pharmacotherapy.

There is evidence that cancer patients may benefit from psychotherapeutic approaches such as cognitive restructuring–based cognitive behavioral therapy (CBT) and problem-solving therapy [4, 5]. However, quite a few cancer patients are reluctant to directly face their concerns regarding death, symptom burden, loss of control, and other factors because their experiences are psychologically traumatic. On the other hand, it has also been reported that cancer patients rate physical activity and other non-standard treatments as helpful for their mental health [6]. Thus, we assume that for some cancer patients who are reluctant to talk about their concerns, it may be feasible to alleviate depressive symptoms by focusing on increased participation in daily activities, since such participation tends to be decreased by depressive patients.

Behavioral activation therapy (BAT) directly addresses daily activities that individuals value most highly. The mechanisms underlying BAT are as follows: (i) individuals suffering from depressive or anxiety conditions experience avoidance and decreased participation in normal activities; (ii) this leads to decreased opportunities for them to experience joy; (iii) they feel that they are overwhelmed by hardships; (iv) the value they place on their lives and themselves is diminished; (v) they pay more attention to negative information; (vi) they eventually experience more distress and depression; (vii) BAT facilitates activities they value and breaks this vicious cycle; and (viii) this improves the depressive condition and (ix) leads to improvement in quality of life. Of note, BAT emphasizes identifying values as a part of behavioral change [7]. Thus BAT, unlike cognitive restructuring–based CBT, is not a cognitive approach and may therefore be useful for cancer patients who are reluctant to talk about their concerns.

The effectiveness of BAT for depression and anxiety is well established [8, 9]. However, there is insufficient evidence of BAT specifically for cancer patients, as only one pre-post study demonstrated improvement [10]. Therefore, to confirm the effectiveness of BAT in cancer patients, we are planning to perform a protocol study of a BAT program in this population. This study was done to confirm the potential of our BAT program for the depression and anxiety of cancer patients.

## Methods

### Setting and subjects

The medical records of the patients were investigated retrospectively. The inclusion criteria were as follows: cancer patients who 1) visited the psycho-oncology division of the National Cancer Center Hospital (NCCH) in Japan, as outpatients or inpatients, from October 2016 to March 2018; 2) met the criteria for Major Depressive Disorder (MDD) or Adjustment Disorders according to the Diagnostic and Statistical Manual of Mental Disorders (5th ed. [DSM-V]; American Psychiatric Association, 2013); and 3) participated in the NCCH BAT program.

This study was approved by our institutional review board. We obtained written consent from all participants.

### Outcome measure

The primary outcome was the BAT program completion percentage (the number of subjects who completed all eight sessions divided by the number of subjects who participated in the program, multiplied by 100). Secondary outcomes were the self-reported depression severity (Patient Health Questionnaire-9 (PHQ-9) score), anxiety disorder status (Generalized Anxiety Disorder-7 (GAD-7) score), and Clinical Global Impression-Improvement (CGI-I) score after the program.

The PHQ-9 is the nine-item depression module from the full PHQ, and a Japanese version has been validated [11]. The nine items pertain to the DSM criteria for MDD as experienced during the 2 weeks prior to and including the day of survey completion. Each item is rated on a 4-point scale from 0 to 3 (0, never; 1, several days; 2, more than half the time; and 3, nearly every day). The total score ranges from 0 to 27, and a score of 11 or greater represents depressive symptoms of at least moderate severity and is the cut-off point for MDD screening in Japan [11].

The GAD-7 is a seven-item questionnaire developed to identify probable cases of GAD and to measure the severity of GAD symptoms; a Japanese version has been validated [12]. The GAD-7 assesses the most prominent diagnostic features of GAD (diagnostic criteria A, B, and C from the DSM-IV). Response categories are “never,” “several days,” “more than half the days,” and “nearly every day,” scored as 0, 1, 2, and 3, respectively. The total score of the GAD-7 ranges from 0 to 21. Among primary care patients and the general population, the GAD-7 has demonstrated good internal consistency, test-retest reliability, and convergent, construct, criterion, and factorial validity. The severity of anxiety symptoms based on the GAD-7 score is assessed as follows: 0–4, none; 5–9, mild; 10–14, moderate; and 15–21, severe [13].

The CGI-I scale ranges from 1 to 7 points and is scored using the following benchmarks: 1, very much improved; 2, much improved; 3, minimally improved; 4, no change; 5, minimally worse; 6, much worse; and 7, very much worse [14]. A correlation has been previously reported between the CGI and generally used rating scales, such as the Hamilton Depression Rating Scale (HRSD), the Montgomery Åsberg Depression Rating Scale, and the Beck Depression Inventory [15]. The severity of major depressive symptoms was assessed using the HRSD score, as follows: 0–7, no depression; 8–16, mild depression; 17–23, moderate depression; and ≥ 24, severe depression [16].

### Procedure of the BAT program

The BAT program was one of the support programs provided by the patient support research and development center in NCCH. The center was opened in September 2016 to provide cancer patients with rehabilitation and social work services, psychological care, nursing consultation, and counseling on nutrition and appearance. The BAT program was a clinical activity available to individuals who met the following criteria: 1) were cancer patients visiting the NCCH in Japan; 2) were over 20 years old; 3) had an Eastern Cooperative Oncology Group performance status (PS) score of 0 to 1, making it easier to modify behaviors; 4) could speak Japanese; and 5) expressed their desire to participate in the program. Exclusion criteria were as follows: 1) serious physical symptoms that prevented participants from finishing the program; 2) cognitive functional disorders and disturbance of consciousness that made it impossible to understand the program content; 3) severe mental symptoms, such as those related to depression, that were accompanied by psychotic symptoms or imminent suicidal ideation, because these indicated the need for more intensive approaches such as pharmacological treatment or electroconvulsive therapy (ECT); and 4) the physicians in charge judged it difficult for their patients to participate in the program.

Each patient completed the PHQ-9 and GAD-7 (before and after the BAT program. We administered the CGI-I scale after the program. We did not limit medication use or adjust medications because some patients required medications in conjunction with psychotherapy for the treatment of depression, and this combination has been reported to be more effective than either modality alone for the treatment of depression [17].

We notified patients of the BAT program via the NCCH homepage and via posters placed throughout the hospital. Moreover, we informed physicians at the NCCH about the BAT program through a hospital seminar and via the NCCH website, and we asked them to introduce the program to their patients. After we checked whether patients met the eligibility and exclusion criteria, we gave them a detailed explanation of the program and the opt-out procedure, then asked if they would like to participate.

### Components of the BAT program

The BAT program consisted of eight 50-min sessions conducted over 1 or 2 weeks, with an average of 5 to 10 min of homework per day. Regular treatment was not affected by the intervention, while special psychotherapies such as BAT (conducted elsewhere) and CBT were prohibited. The outlines of the BAT program are shown in Table 1.

**Table 1:** Outline of the Behavioral Activation Therapy Program

	Theme	Aims/Contents
1	Let’s begin	Understand the relationship between emotions and behaviors
		Learn tips on how to avoid being preoccupied by cancer in order to regain pleasure and meaning in life
2	Identify the relationship between emotions and behaviors	Explore the relationship between emotions and daily activities
		Identify patterns of anxiety and depression, and patterns of feeling calm
3	Identify activities that make your life pleasurable	Clarify values in life
		Identify activities that are achievable in your current situation
4	Review the results of activities	Evaluate the usefulness and difficulty of each activity
		Discuss ways to participate in activities that are challenging to engage in
5	Identify difficult situations	Identify situations that are likely to lead to anxiety and depression
		Identify thoughts that occur in the early stages of anxiety and depression, and any vicious cycle that may be involved
6	Understand ways to change your feelings	Identify pros and cons of negative thinking
7	Learn to live a life of value	Review how the program helped change patterns of daily life
		Identify future goals and make plans to achieve them
8	Wrap-up and graduation	Review what has been learned through the program
		Identify future goals and make plans to achieve them

### Therapists

The supervisor of the program was a professor specializing in BAT and CBT. He led eight sessions, all of which were attended by a psychiatrist and two psychologists who work at our hospital. All therapists had at least 5 years of clinical experience that included work with cancer patients. After attending the supervisor’s sessions, the psychiatrist and psychologists conducted the program on their own under supervision.

### Analysis

We analyzed both demographic data and the completion percentage by descriptive statistics, and both depression and anxiety by the matched paired t-test.

## Results

Ten patients participated in the BAT program during the study period. Of them, nine (age, 57.8 years (SD, 9.6)) completed the program, resulting in a completion percentage of 90%. One patient dropped out of the program due to cognitive impairment secondary to brain metastasis.

Patient characteristics are shown in Table 2. There were nine females and one male. Cancer types were breast cancer in six patients, and lung cancer, laryngeal cancer, endometrial cancer, and ovarian cancer in one patient each. The psychiatric diagnosis was MDD in seven patients and adjustment disorders in three patients. Both the PHQ-9 scores (pre: 14.4 (SD, 6.1); post: 5.1 (SD, 5.8)) and the GAD-7 scores (pre: 11.9 (SD, 4.9); post: 4.7 (SD, 5.5)) were significantly improved after the BAT program (Table 3). The CGI-I scores ranged from 1 to 3, with all patients showing clinical improvement except the one who dropped out (Case No. 3).

**Table 2:** Patient Characteristics

No.	Age	Sex	Cancer type	Stage	Treatment	Psychiatric diagnosis	Antidepressant/ Stabilizer	Medication adjustment	PHQ-9		GAD-7		CGI-I	Complete or
									pre	post	pre	post		Drop out
1	45	F	Breast cancer	II	Chemotherapy	Depression (moderate)	Escitalopram	Yes (discontinue)	15	2	8	4	2	Complete
2	62	F	Lung cancer	IV	Chemotherapy	Depression (moderate)	Escitalopram Lorazepam	Yes (increase)	15	1	15	2	1	Complete
3	48	F	Breast cancer	IV	Chemotherapy	Depression (moderate)	Mirtazapine	Yes (new prescription)	13	×	3	×	×	Drop out (brain metastasis)
4	66	F	Endometrial cancer	III	Chemotherapy	Depression (severe)	Escitalopram Alprazolam	Yes (increase)	19	19	19	18	3	Complete
5	66	F	Ovarian cancer	Recurrence	Chemotherapy	Depression (moderate)	Nothing	No	14	2	8	0	1	Complete
6	72	F	Breast cancer	IV	Chemotherapy	Adjustment disorder (with anxiety)	Nothing	No	6	1	6	0	1	Complete
7	56	F	Breast cancer	I	Observation	Adjustment disorder (with anxiety)	Nothing	No	6	3	10	4	2	Complete
8	57	F	Breast cancer	II	Chemotherapy	Adjustment disorder (with anxiety)	Ethyl loflazepate Alprazolam	No	20	4	18	6	1	Complete
9	62	M	Laryngeal cancer	Recurrence	Observation	Depression (moderate)	Escitalopram Ethyl loflazepate	No	24	9	15	6	1	Complete
10	44	F	Breast cancer	II	Chemotherapy	Depression (moderate)	Nothing	No	11	5	8	2	2	Complete

**Table 3:** Changes in PHQ-9 and GAD-7 scores

		pre			post		
	M		SD	M		SD	*P* value
PHQ-9	14.4		6.1	5.1		5.8	0.0014
GAD-7	11.9		4.9	4.7		5.5	0.0004

## Case presentation

The clinical course varied among the ten patients who participated in the BAT program. Most cases showed clinical improvement, however, there was one case with poor clinical improvement. Here, we report three cases with distinctive results.

### Case 1

A 45-year-old housewife who lived with her husband and two children who was diagnosed as having breast cancer (Stage II) and received a surgical resection. After surgery, she was told that she would need adjuvant chemotherapy. She became anxious about the possible recurrence of her breast cancer and side effects of chemotherapy such as hair loss and nausea. Consequently, she could not go out and stayed home all day long. She gradually became depressive, so she visited the psycho-oncology division of our hospital. She was diagnosed as having an MDD and prescribed antidepressant. However, she was unable to continue the medication because of nausea and fatigue. Though another antidepressant was proposed, she did not agree to use it because of concerns about the side effects. Therefore, she participated in the BAT program. At the beginning of the program, her depression was considered to be moderately severe by HRSD. However, she achieved remission of depression by the end of the program, without antidepressants, and began to live an active life.

### Case 4

A 66-year-old housewife who lived with her husband was found during a biannual medical checkup to have endometrial cancer (Stage III). She was so shocked that she became seriously depressed. She repeatedly thought, “why do I have cancer despite a biannual medical checkup”“I should have had more medical checkups, if I had, I might have no cancer.” She became very nervous and anxious about the possible recurrence of her cancer and its development into a serious physical disease. Her psychiatrist prescribed an antidepressant. She subsequently began participating in the BAT program. However, she was absent-minded during much of every session, and her compliance with the assigned daily homework was poor. Although antidepressant was increased, her depression did not improve much.

### Case 9

A 62-year-old man who lived with her wife and had his own business was diagnosed as having laryngeal cancer recurrence and had an operation 2 years previously. He was only given followed up care and had no symptoms. However, he became nervous about the possible recurrence of his cancer. He repeatedly thought, “cancer may spread to other parts of my body”, “if so, I may die” and he kept to his room all day long. He gradually became depressive, so he visited the psycho-oncology division of our hospital. He was diagnosed as having an MDD and prescribed antidepressant. He subsequently began participating in the BAT program. After taking part in the program, increased business activity, on which he placed great value, gradually allowed him to live his life actively without concerns.

## Discussion and conclusions

The completion percentage in the BAT program was 90% (nine out of 10 patients), and the only patient who did not complete it dropped out due to cognitive impairment secondary to brain metastasis. These results suggest that the program is feasibly for use with cancer patients.

All patients except the one who dropped out demonstrated clinical improvement according to the CGI-I. Furthermore, both PHQ-9 and GAD-7 scores were significantly improved after the program. These results suggest that the BAT program is effective for patients with cancer.

From the cases with good clinical courses (Case 1 and Case 9), we are of the view the BAT program would be effective in cases where patient has recurrent anxiety and rumination and their activity decreases due to them. BAT facilitates activities they value and brings about positive reinforcement for the patients. As a result, BAT breaks the vicious cycle which occurs from recurrent anxiety and rumination and leads to improvement of the depressive condition. Both cases suggest that BAT can lead patients to live independent and active lives after their cancer diagnosis. Furthermore, Case 1 suggests that BAT can be adapted particularly well to cancer patients who do not want to take antidepressants because of concerns about side effects.

Questionnaire results of the patient with poor clinical improvement (Case No. 4) showed slight decreases in cancer-related distress and anxiety about the possibility of recurrence, but overall the effect of the program was not significant. Given her inadequate response to the program, as well as previous findings that the effects of BAT are poor in patients with severe depression and poor compliance [18], BAT programs may be ineffective for cancer patients with severe depression, in part because the condition leads to poor compliance.

This study has several limitations. First, it used a retrospective design that may have caused several systematic biases. Second, it was conducted at a single cancer center, raising the question of institutional bias, and the results may not be applicable to other settings. Third, there is a possibility of assessment bias. Although psychiatric diagnoses were made using DSM-V criteria, this method is not robust because our diagnoses were clinical in nature and were not based on the Structured Clinical Interview for DSM-5 (SCID-5). Moreover, the CGI-I is a subjective scale and does not have a specific anchor point. Fourth, the study sample was small (*n* = 10). According to one report, pilot studies should have sample sizes of at least 30 [19]. The BAT program may not have been advertised adequately, because only 14 patients participated during the study period, including four with a psychiatric diagnosis other than MDD and adjustment disorders. Fifth, the effectiveness of BAT should not be exaggerated given the possibility of bias, for instance due to the effect of pharmacotherapies and other co-interventions (support from medical staff and their family members).

Despite its limitations, this case series study suggests that BAT is potentially effective for the depression and anxiety of cancer patients. To establish further evidence for BAT use with cancer patients, randomized controlled trials are needed.

## Abbreviations

BAT: Behavioral activation therapy
CBT: Cognitive behavioral therapy
CGI-I: Clinical Global Impression-Improvement
DSM: Diagnostic and Statistical Manual of Mental Disorders
ECT: Electroconvulsive therapy
GAD-7: Generalized Anxiety Disorder-7
HRSD: Hamilton Depression Rating Scale
NCCH: National Cancer Center Hospital
PHQ-9: Patient Health Questionnaire-9
SCID: Structured Clinical Interview for DSM
SD: Standard deviation
